# Psilocybin for Treating Psychiatric Disorders: A Psychonaut Legend or a Promising Therapeutic Perspective?

**DOI:** 10.3390/jox12010004

**Published:** 2022-02-07

**Authors:** Maurizio Coppola, Francesco Bevione, Raffaella Mondola

**Affiliations:** 1 Department of Addiction, ASL CN2, Corso Coppino 46, 12051 Alba, CN, Italy; francescobevione@gmail.com; 2 Department of Mental Health, ASL CN1, Via Torino 70/B, 12037 Saluzzo, CN, Italy; raffaellamondola@virgilio.it

**Keywords:** psilocybin, psilocin, psychedelics, magic mushrooms

## Abstract

Psychedelics extracted from plants have been used in religious, spiritual, and mystic practices for millennia. In 1957, Dr. Hofmann identified and synthesized the prodrug psilocybin, a substance present in more than 200 species of psychedelic mushrooms. Although there were limitations related to the scientific design of many studies, clinical observations performed during the 1950s and 1960s showed a potential therapeutic effect of psilocybin for patients affected by depressive symptoms, anxiety, and conversion disorder. Psilocybin was classed as a schedule I substance in 1970, but the fascination with psychedelics has remained almost unchanged over time, promoting a new scientific interest starting in the 1990s. Recent studies have provided further evidence supporting the suggestive hypothesis of the therapeutic use of psilocybin for treating various psychiatric disorders, including pathological anxiety, mood depressive disorder, and addiction.

## 1. Introduction

Psychedelics extracted from plants have been used in religious, spiritual, and mystic practices for millennia [1]. The use of peyote cactus buttons and red beans containing mescaline by humans has been documented for 5700 years in the northeastern region of Mexico [2]. The analysis of archaeological artifacts has confirmed that the use of psilocybin-containing mushrooms has been ubiquitous since prehistory [3]. The first report of the use of psychedelic mushrooms in Western medicine was made by Prentiss and Morgan in 1895. The authors described the ceremonial use of peyote cactus buttons by indigenous people in Central America [4]. Mescaline, an active alkaloid contained in peyote, was isolated by Arthur Heffter in 1897 and synthesized by Ernest Spath in 1919. Subsequently, it was made available as a research chemical by the Merck & Co. pharmaceutical company [5]. In 1938, at the Sandoz laboratories in Switzerland, Albert Hofmann synthesized lysergic acid diethylamide, best known as LSD. This substance was synthesized during a systematic study investigation of ergot alkaloids in which LSD was the 25th compound produced. In 1947, LSD was marketed under the trade name “Delysid” and was made freely available to researchers interested in investigating its pharmacological properties [6]. In 1957, Dr. Hofmann also identified and synthesized the prodrug psilocybin, a substance present in more than 200 species of psychedelic mushroom. In 1958, psilocybin was made available by Sandoz under the brand name “Indocybin”. During the 1950s and 1960s, psilocybin, LSD, and mescaline were largely used for treating non-psychotic disorders. In more than 1000 scientific reports, authors described the results obtained from the treatment of about 40,000 patients [7]. Although there were limitations related to the scientific design of many studies, clinical observations performed during the pre-prohibition era showed a potential therapeutic effect of psilocybin for patients affected by depressive symptoms, anxiety, and conversion disorder [8,9,10,11,12]. Contrariwise, there is very limited information about the therapeutic effects of psilocybin in psychotic patients [13,14]. On the whole, patients treated with psycholytic or psychedelic doses of psilocybin reported no significant side effects [15]. In the 1960s, psychedelics became widely used as recreational drugs, as well as a symbol of the counterculture. Most human studies reported low toxicity; however, some severe psychiatric reactions and occasional tragic events reported in the scientific literature produced socio-political alarm in many countries [16,17]. Consequently, the interest in medical research for studying the potential therapeutic activity of psychedelics was reduced, and these substances became considered unethical for medical use [17]. Psilocybin was classed as a schedule I substance in 1970, but the fascination with psychedelics remained almost unchanged over time, promoting a new scientific interest starting from the 1990s [17,18]. Recent studies have provided further evidence supporting the suggestive hypothesis of the therapeutic use of psilocybin for treating various psychiatric disorders, including pathological anxiety, mood depressive disorder, and addiction [17,18]. In our review, we summarize the clinical, pharmacological, and toxicological information currently available about psilocybin, focusing our attention on evaluating the therapeutic effects in humans.

## 2. Chemistry

**Figure 1:**
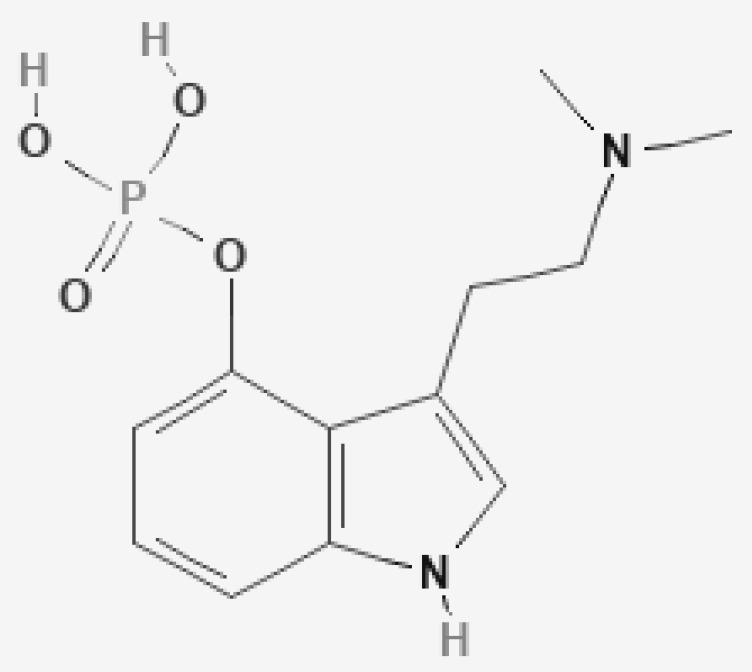
Psilocybin. PubChem: https://pubchem.ncbi.nlm.nih.gov/compound/10624#section=2D-Structure (accessed on 10 June 2021).

**Figure 2:**
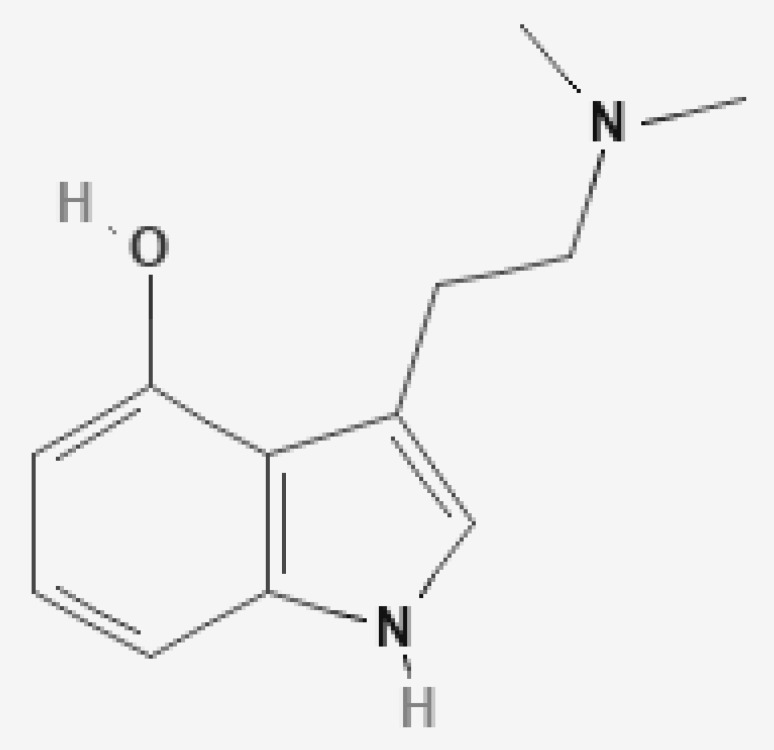
Psilocin. PubChem: https://pubchem.ncbi.nlm.nih.gov/compound/4980#section=2D-Structure (accessed on 10 June 2021).

Psilocybin (Figure 1) and psilocin (Figure 2) are tryptophan indole-based compounds present in mushrooms of the genus *Psilocybe, Panaeolina, Pluteus, Panaeolus, Stropharia, Conocybe, and Gymnopilus.* These mushrooms are known and distributed worldwide [19,20,21]. Their indole ring structure derives from a fusion between a pyrrole ring and a benzene ring, joined to an amino group by a two-carbon side chain [22]. Psilocybin, IUPAC name [3-[2-(dimethylamino)ethyl]-1H-indol-4-yl] dihydrogen phosphate, is a tertiary amino compound belonging to the tryptamine alkaloid group. This substance, with a molecular weight of 284.25 g/mol, has a phosphoryloxy substituent attached at position four of the N,N-dimethyltryptamine structure. Psilocin, IUPAC name 3-[2-(dimethylamino)ethyl]-1H-indol-4-ol, is the dephosphorylated psilocybin derivative representing the active compound of psilocybin. Psilocin, molecular weight 204.27 g/mol, is a tryptamine alkaloid in which an additional hydroxy group is attached to the N,N-dimethyltryptamine skeleton. Psilocybin has a water solubility of 2.7 g/L and a melting point of 224 °C. Psilocin has a water solubility of 4.08 g/L and a melting point of 174.5 °C [23]. Psilocybin is a zwitterion alkaloid with a highly polar phosphate group; consequently, it is more soluble in water than psilocin [24]. Contrariwise, psilocin is more lipid soluble than psilocybin. Both substances are soluble in methanol and ethanol but almost insoluble in ether, chloroform, and petroleum. In pure form, psilocybin and psilocin are white crystalline powders, unstable in light but relatively stable under an inert atmosphere, in the dark, and at low temperatures [23,24].

## 3. Pharmacokinetics

Magic mushrooms are usually taken orally or, less frequently, smoked. The concentrations of psilocybin and psilocin in psychedelic mushrooms are 2% and 0.5%, respectively [25]. However, the concentrations of active principles may vary in relation to the species, origin, size, age, growing, and drying conditions [26]. In animal studies, it was found that after oral administration, 50% of the 14C-labelled psilocin was absorbed and almost evenly distributed throughout the body, including the brain [27]. In pregnant rat studies, after intravenous administration, the 14C-psilocin crossed the placental barrier reaching fetal tissue concentrations lower than maternal tissue, but with a slower elimination half-life [28]. In adult men, following oral administration, psilocybin is dephosphorylated to psilocin from the hydrochloric acid made by the stomach [29,30]. Furthermore, psilocybin is dephosphorylated to psilocin in the intestine, kidney, and blood by the alkaline phosphatase and nonspecific esterases [29,30]. In rat studies, it was found that psilocybin was more easily absorbed from the jejunum and colon than psilocin [31]. Moreover, many other rodent tissues can convert psilocybin to psilocin before the transit into the systemic circulation [31]. In rat studies performed with the 14C-labelled psilocybin, psilocin crossed the blood–brain barrier and entered the central nervous system, where it exerted its psychotropic effect [32]. In humans, if administered in the empty stomach, psilocybin is rapidly converted to psilocin, which is detectable in the plasma within 20–40 min [29]. Maximum psilocin plasma concentrations are reached within 80–100 min [29]. Since psilocin is structurally related to the neurotransmitter serotonin, it follows a comparable human metabolism [33]. In fact, about 4% of psilocin is metabolized by demethylation and oxidative deamination, catalyzed by the liver monoamine oxidase (MAO) or aldehyde dehydrogenase, via a presumed intermediate metabolite, 4-hydroxyindole-3-acetaldehyde, to yield 4-hydroxy-indole-3-acetic acid, 4-hydroxy-indole-3-acetaldehyde, and 4-hydroxytryptophole [34,35]. After oral administration, the plasma elimination half-lives estimated for psilocybin and psilocin are 160 and 50 min, respectively [29,36]. In rat studies, after oral administration, it was found that psilocin was excreted in the urine at 65%, and in the bile and feces at approximately 15–20% within 8 h [34,35,36,37,38]. In rat studies, about 25% of the whole psilocybin dose was excreted unaltered, whereas about 10–20% remained in the body, with its metabolites detected in the urine for 6–7 days [34]. In a study performed on male volunteers, around 3.5% of the oral psilocybin dose was excreted in the urine as free psilocybin within 24 h [29,36]. As emerged in pharmacokinetic and forensic studies, approximately 80% of psilocin is eliminated as psilocin-O-glucuronide [37,38]. In the small intestine, glucuronidation is mediated by the glucuronosyltransferase UGT1A10 [39]. Instead, when psilocin is administered intravenously, glucuronidation is mediated by the glucuronosyltransferase UGT1A9 [39]. Conversely, N-glucuronidation was not observed in cell studies [39]. Finally, the third metabolic pathway might be the oxidation of psilocin by the hydroxyindole oxidases to produce compounds with an o-quinone or iminoquinone structure [40].

## 4. Pharmacodynamic

Psilocybin and psilocin exert a predominant agonist activity at serotonin receptors, particularly the 5HT2A receptor. Agonist activity at the 5HT2A receptor is generally considered a key pharmacological mechanism for inducing hallucinogenic effects. The role of other receptors is documented, but less investigated [41]. In all studies, psilocin displayed high 5HT2A receptor affinity (ki = 6 nM). In addition, psilocin binds many other serotonin and non-serotonin receptors including: 5HT2B; 5HT1D; D1; 5HT1E; 5HT1A; 5HT5A; 5HT7; 5HT6; D3; 5HT2C; 5HT1B. A weak imidazoline 1, alpha 2A, alpha 2B, alpha 2C receptors, and 5HT transporter affinity was also demonstrated [42]. Unlike LSD, there was no information showing the pharmacodynamic activity of psilocin at the D2 receptor [43]. In human studies, pre-treatment with the 5HT2A receptor antagonist ketanserin blocked the psychotomimetic effects of psilocybin in a dose-dependent manner [44]. Furthermore, psychotomimetic effects were also blocked using a pre-treatment with the atypical antipsychotic risperidone [44]. On the contrary, psychotomimetic effects were increased by the dopamine antagonist and typical antipsychotic haloperidol. In line with this result, psilocybin could exert its psychotropic effect with a mechanism of action independent/partially independent from dopamine stimulation [44]. However, in a positron emission tomography (PET) study performed on male volunteers using the D2 dopamine receptor antagonist [11C]-raclopride, psilocybin decreased the [11C]-raclopride receptor-binding bilaterally in the caudate nucleus (19%) and putamen (20%). These results suggest an increase in endogenous dopamine in response to psilocybin administration. In humans, changes in the [11C]-raclopride receptor-binding in the ventral striatum have been correlated with depersonalization and euphoria; consequently, 5-HT1A and 5-HT2A receptor stimulation could be important for striatal dopamine release. Psychotropic effects induced by psilocybin could be related to both striatal dopamine release and serotonin transmission [45]. In human studies, equimolar amounts of psilocybin and psilocin induced the same psychotropic effects [46]. However, the inhibition of dephosphorylation using the alkaline phosphatase competitive antagonist beta-glycerophosphate prevented all symptoms induced by psilocybin. This clinical information has strongly confirmed that psilocin is the main active metabolite, and responsible for the psychedelic effects experienced [47].

## 5. Functional Studies

Electroencephalographic alterations induced by psilocybin in humans and animal models have been studied since the 1960s [48,49,50,51,52]. The first electroencephalographic studies performed in primates and humans under psilocybin intoxication showed numerous electroencephalographic tracing alterations, such as a decrease in alpha and theta activity, an increase in fast activity, and desynchronization [48,49,50,51,52]. Changes in visually evoked potentials were described in humans [51,52]. In a visual-evoked potentials study performed on 26 healthy male volunteers, psilocybin decreased prestimulus parieto-occipital α-power values, precluding a subsequent stimulus-induced α-power decrease. Moreover, psilocybin decreased N170 potentials that were associated with visual perceptual alterations, including visual hallucinations. All effects were blocked by pre-treatment with the 5-HT2A antagonist ketanserin [53]. In a magnetoencephalography study performed on a group of fifteen healthy male volunteers, after the intravenous infusion of psilocybin, a spontaneous cortical oscillatory power reduction from 1 to 50 Hz in the posterior association cortex was found, and from 8 to 100 Hz in the frontal association cortex. Conversely, no effect was found on low-level visually induced or motor-induced gamma-band oscillations. Dynamic causal modelling showed a correlation between posterior cingulate cortex desynchronization and increased excitability of the deep-layer pyramidal neurons. This correlation appeared to be triggered by the 5-HT2A receptor-mediated excitation of deep pyramidal cells [54]. In a PET and [F-18]-fluorodeoxyglucose (FDG) study performed on 10 healthy volunteers, prior to and following a 15 or 20 mg dose of psilocybin, authors found a global increase in the cerebral metabolic rate of glucose with a predominant localization in the frontomedial and frontolateral cortex, anterior cingulate, and temporomedial cortex. Instead, a smaller increase in the metabolic rate of glucose was found in the basal ganglia, sensorimotor area, and occipital cortex [55]. In a double-blind, placebo-controlled study performed on healthy volunteers using the [F-18]-fluorodeoxyglucose FDG PET, psilocybin increased the metabolic rate of glucose in the right anterior cingulate, right frontal operculum, and right inferior temporal region. Conversely, a significant decrease in the metabolic rate of glucose was found in the right thalamus, left precentral region, and left thalamus. Authors have further observed a trend decrease in the metabolic rate of glucose in the composite right hemisphere and bilateral subcortical regions, as well as a trend increase in the cortical/subcortical ratio of the right hemisphere [56]. Carhart-Harris et al. designed a functional MRI study to capture the transition from normal waking consciousness to the state induced by the intravenous infusion of 2 mg of psilocybin. Arterial spin labelling perfusion and a blood–oxygen level-dependent functional MRI were used to map cerebral blood flow and changes in venous oxygenation before and after the placebo and psilocybin infusion. Results showed a significant cerebral blood flow (CBF) decrease in the subcortical (bilateral thalamus, putamen, and hypothalamus) and cortical regions (posterior cingulate cortex (PCC), retrosplenial cortex, precuneus, bilateral angular gyrus, supramarginal gyrus, rostral and dorsal anterior cingulate cortex (ACC), paracingulate gyrus, medial prefrontal cortex (mPFC), frontoinsular cortex, lateral orbitofrontal cortex, frontal opercu-lum, precentral gyrus, and superior, middle and inferior frontalgyrus). Subjective effects were strongly related to the decreased activity and connectivity in the brain’s key connector hubs including thalamus, mPFC, and ACC [57]. In their placebo-controlled, double-blind study—performed to measure perfusion changes, with and without adjustment for global brain perfusion, after two doses of oral psilocybin (low dose: 0.160 mg/kg; high dose: 0.215 mg/kg)—in two groups of healthy volunteers, Lewis et al. showed a reduction in absolute perfusion in the frontal, temporal, parietal, and occipital lobes, bilateral amygdalae, anterior cingulate, insula, striatal regions, and hippocampi. The data that emerged from the study suggest that relative changes in brain perfusion should be interpreted in relation to the absolute signal variations and analysis method [58]. In a psilocybin vs. placebo cross-over functional MRI study, psilocybin enhanced the autobiographical recollection facilitating the underlying neural processes. Significant activation was found in the limbic and striatal region in the early phase. Otherwise, significant activation in the late phase was found in the medial prefrontal cortex. Additional visual and sensory cortical activation in the late phase was found under psilocybin only. Rating of memory vividness and visual imagery was significantly higher after psilocybin than placebo. Furthermore, authors found a significant positive correlation between vividness and subjective well-being at follow-up [59]. In a PET study performed on eight healthy volunteers using the 5-HT2A receptor agonist radioligand [11C]-Cimbi-36, oral intake of 3–30 mg of psilocybin produced a dose-related 5-HT2A receptor occupancy. Moreover, the study highlighted a correlation between subjective effects induced by psilocybin, 5-HT2A receptor occupancy, and plasma psilocin levels [60]. In two PET studies performed on healthy volunteers using the 5-HT2A receptor agonist radioligand [11C]-Cimbi-36, after psilocybin administration, individual brain 5-HT2A receptor-binding predicted subjective mystical effects [61], mindfulness, and openness [62].

## 6. Toxicity

Psilocybin is generally considered to be well tolerated and low in toxicity. Some cases of fatal intoxication have been reported; however, the majority of them were not directly linked to the toxic effects induced by psilocybin. They were related to mixed drug intoxication, suicide, and jumping out of the window during hallucinations [26,63]. In 1996, Gerault and Picart described a case in which a massive dose of psilocybe semilanceata was considered as the cause of death. Toxicological examination evidenced a psilocin plasma level of 4 µg/mL [64]. The human lethal dose is not known; however, the LD50 for a rat, mouse and rabbit, after the intravenous administration of psilocybin, were 280 mg/kg, 275 mg/kg, and 13 mg/kg, respectively [65]. In comparison, the LD50 for a rat, mouse and rabbit, after intravenous administration of psilocin, were 75 mg/kg, 74 mg/kg, and 7 mg/kg, respectively [66]. The human toxic dose low (TDLo) for oral psilocybin administration was 0.04–0.06 mg/kg, whereas TDLo for intravenous psilocybin administration was 1–2 mg, corresponding to a psilocin plasma level of 4–6 ng/mL. At these dosages, patients reported visual field changes, muscle weakness, nausea, and vomiting. In dose-effect studies, psilocybin was found to be 66 times more potent than mescaline and 45 times less potent than LSD [67] In two cross-over studies performed at the end of the 1950s, authors found cross tolerance between psilocybin and LSD [68]. Psilocybin is principally used for its psychedelic effects, including altered self-perception, impaired perception of time and space, alteration in thought contents, derealization, depersonalization, body image distortion, and alterations in mood and emotions [69,70,71]. As previously reported, symptoms induced by psilocybin can be reverted using the 5HT2A/C antagonist ketanserin or 5HT2A/C and D2 antagonist risperidone. Haloperidol, a D2 antagonist, can normalize euphoria, derealization and depersonalization [44]. On the other hand, MAO inhibitors can intensify psychedelic effects induced by psilocybin [72]. Alcohol can enhance the psychedelic effects induced by psilocybin, since its metabolite acetaldehyde reacts with the endogenous biogenic amines producing the MAO inhibitors tetrahydroisoquinoline and β-carbolines [73]. Psilocybin effects can be prolonged by tobacco, because it may reduce the central nervous system and peripheral tissue MAO B levels [74]. In addition to the central nervous system, psilocybin can affect other organs and systems, including the renal [75], cardiovascular, respiratory, gastrointestinal, visual, and musculoskeletal systems [76], as reported in Table 1. Overall, psychotropic and neuropsychological effects appear to be influenced by personal expectations, setting, and brain structure metrics [41,77]. Prolonged hallucinations or psychotic experiences are rarely reported in healthy persons, when compared with people affected by psychotic or personality disorders [78] However, long-lasting unpleasant experiences, best known as “bad trips” or hallucinogen-persisting perception disorder (HPPD), have been reported [79]. Psilocybin does not directly affect the mesolimbic dopaminergic pathway involved in the reward system; consequently, it does not induce craving, addiction or withdrawal [41,76]. Finally, there is not enough information to confirm or exclude genotoxicity or teratogenicity [80].

## 7. Psilocybin and Mood Disorders

In a double-blind, placebo-controlled study performed on 12 patients (11 women and 1 man), affected by advanced-stage cancer, 0.2 mg/kg of psilocybin administered in a single dose produced a significant reduction in anxiety at 1 and 3 months, and depressive symptoms at 6 months, compared with the placebo (niacine 250 mg). Symptoms of anxiety and depression were assessed using the State-Trait Anxiety Inventory and Beck Depression Inventory [81]. In a two-session, double-blind cross-over study, authors compared the effect of low (1 or 3 mg/70 kg) versus high (22 or 30 mg/70 kg) psilocybin dose on depressive symptoms, anxiety, and quality of life in 51 patients with life-threatening cancer. High-dose psilocybin produced a significant decrease in depressive symptoms, anxiety and death anxiety, along with a significant increase in quality of life and optimism. At the 6-month follow-up, improvement in mood, anxiety and quality of life were confirmed in about 80% of the patients [82]. In a similar double-blind, placebo-controlled, cross-over trial performed on 29 patients affected by life-threatening cancer, a single-dose psilocybin of 0.3 mg/kg improved depressive symptoms, anxiety and quality of life in the weeks after administration. At the 6.5-month follow-up, around 80% of patients had kept these clinical benefits [83]. In an open-label study performed on 12 patients (6 men and 6 women), affected by moderate-to-severe unipolar, treatment-resistant major depression, two oral doses of psilocybin (10 mg and 25 mg, 7 days apart) in association with psychological support, before, during, and after each session, produced a marked reduction in depressive symptoms, as assessed by the 16-item Quick Inventory of Depressive Symptoms (QIDS) at 1 week and 3 months. Patients reported mild adverse effects such as transient headaches, anxiety, confusion, and nausea [84]. In another open-label study performed on 20 patients (12 males and 6 females), affected by severe unipolar, treatment-resistant major depression, two oral doses of psilocybin (10 mg and 25 mg, 7 days apart), in association with psychological support, produced a marked reduction in depressive symptoms, as assessed by the 16-item Quick Inventory of Depressive Symptoms (QIDS) at 1 week, 5 weeks, 3 months, and 6 months. Depressive symptom reduction at 5 weeks was predicted by the quality of the acute psychedelic experience [85]. Recently, two further clinical studies have confirmed the efficacy of psilocybin in patients affected by major depressive disorder. In the first study, 24 of 27 patients completed a randomized, waiting-list-controlled clinical trial at the Johns Hopkins Medical Center. Patients received psilocybin at moderately high (20 mg/70 kg) and high (30 mg/70 kg) doses in two sessions. Statistical analysis showed a significant decrease in GRID-HAMD scores from baseline to weeks 1 and 4 [86]. In the second study, 59 patients affected by moderate to severe major depressive disorder were enrolled in a phase 2, double-blind randomized, controlled trial in which the antidepressant effect of psilocybin was compared to escitalopram. The authors showed no difference between the groups at week 6 in the 16-item Quick Inventory of Depressive Symptomatology-Self-Report (QIDS-SR-16) score [87].

## 8. Psilocybin and Obsessive–Compulsive Disorder

Clinical information regarding the potential therapeutic effects of psilocybin in patients affected by obsessive–compulsive disorder is very limited. In a double-blind study performed on 9 patients (7 males and 2 females), affected by resistant obsessive–compulsive disorder, psilocybin showed to be safe and effective in reducing obsessive–compulsive symptoms for a duration extended beyond the psychedelic effect [88]. Psilocybin was administered in up to four different doses in a modified dose escalation from very low dose (25 μg/kg) to high dose (300 μg/kg) [87]. In 2014, Wilcox described a case report in which a patient had used psilocybin for years in order to reduce obsessive–compulsive symptoms [89]. This case report followed the case report of Leonard and Rapoport, in which authors described the history of a 17 year-old patient who used LSD and psilocybin to reduce obsessive–compulsive symptoms [90].

## 9. Psilocybin and Addiction

In a proof-of-concept study performed on 10 volunteers (6 men and 4 women), oral administration of psilocybin in one or two sessions in combination with motivational enhancement therapy induced a reduction in drinking days during the subsequent 5–12 weeks. Drinking-day reduction was correlated to the mystical quality of psychedelic experience. Patients did not report significant side effects [91]. The first pilot study performed on 15 people (10 males and 5 females)—which involved a smoking cessation program, and psilocybin in combination with cognitive behavioural therapy—induced a seven-day point prevalence abstinence at 6-month follow-up in 12 of the 15 participants [91]. In a similar open-label pilot-study performed on 12 people—which involved smoking cessation treatment, and psilocybin in combination with cognitive behavioural therapy—6 months of abstinence was produced in 80% of volunteers, without significant side effects. In the sample, abstinence was related to the mystical quality of psychedelic experience [92]. An open label study is currently in progress, in which the primary endpoint is the assessment of the safety of concurrent buprenorphine and naltrexone administration. The estimated study completion date is November 2021 [93].

## 10. Discussion

In recent years, there has been a resurgence of scientific interest about the potential use of psilocybin and other psychedelics for treating psychiatric disorders, in particular mood disorders, anxiety and addiction [17,18]. Recent clinical studies have tried to fill the methodological errors presented by the past studies, including the small size of the enrolled samples, absence of double-masking design, non-use of validated tools for measuring the life expectancy of patients, and non-use of biomarkers [94]. As emerged from the 1950s and 1960s studies, psilocybin has been shown to be safe and well tolerated, particularly when used at therapeutic doses. The most commonly reported side effects were anxiety, headaches, nausea, confusion, vomiting, and slight sympathomimetic symptoms [67,68,69,72,73,81]. All symptoms were described as transient, and no patients required any specific pharmacological treatment. In patients affected by mood depressive disorder and anxiety, psilocybin was displayed to be effective in reducing depressive symptom in short-, medium- and long-term analysis [78,79,80,81,82]. Antidepressant activity lasted longer than psychotropic effects; however, the quality of acute psychedelic experience significantly influenced the therapeutic results [82,88]. The most important pharmacological property showed by psilocybin, in all trials, was the rapid onset of the antidepressant effect. This effect could allow an improvement if used in conjunction with traditional antidepressants therapy, which has a long latency of action [90]. However, no study compared psilocybin with other rapid-acting antidepressants such as ketamine. On the other hand, our analysis of information extracted by clinical studies performed on patients affected by depression has shown two principal limitations: first, the small size of the enrolled samples; second, the comorbidity between depressive symptoms and severe diseases (cancer) in many patients. Overall, the studies have only enrolled a few dozen patients; therefore, results cannot be generalized for more heterogeneous people in terms of age, social status, and disease duration. Moreover, depressive symptoms associated with other diseases could have different expression/evolution compared with primary mood depressive disorder. Consequently, the pharmacological response to therapeutic dosages of psilocybin could be different in primary and secondary depression. In addition, no study included personal expectancy measures as a concomitant variable in the statistical analyses of clinical responses. The unblinding design, expectancy of participants, and evaluators, could be in part responsible of the good results found in all clinical studies. Further and more robust trials are needed to better understand the potential therapeutic properties of this psychedelic. Recently, in a clinical study performed on patients affected by depression, the antidepressant effect of psilocybin was comparable to that of citalopram [87]. Finally, the effectiveness of psilocybin for treating other illnesses, such as obsessive–compulsive disorder and addiction, is not demonstrable, due to the paucity of clinical information currently available. The positive results reported by the authors can be considered interesting hypotheses to be explored in future and more robust clinical studies. In conclusion, psilocybin confirms to be safe and well tolerated when administered at therapeutic doses. Clinical studies currently available, in particular those performed in patients affected by mood depressive disorder, show encouraging therapeutic results requiring further and better designed trials.

## Figures and Tables

**Table 1:** Psilocybin effects [69,70,71].

Central Nervous System	Dream-like state, illusions, hallucinations, synesthesiae, paraesthesia altered state of consciousness, altered self-perception, derealization, depersonalization, altered perception of time and space, altered mood, altered concentration, delusions or unusual ideas, altered emoziona state, euphoria, panic attacks, convulsions, headache, verigo, flushing.
Visual System	Mydriasis
Cardiovascular System	Achicardia, hypertension, hypotension
Respiratory System	Hypoxemia
Gastrointestinal System	Nauseas, vomiting, abdominal pain
Renal System	Urinary incontinence, renal failure
Musculoskeletal System	Muscle weakness

## Data Availability

Not applicable.

## References

[1] Nutt D. (2019). Psychedelic drugs—A new era in psychiatry?. Dialogues Clin. Neurosci..

[2] Bruhn J.G., de Smet P.A., El-Seedi H.R., Beck O. (2002). Mescaline use for 5700 years. Lancet.

[3] Akers B.P., Ruiz J.F., Piper A., Ruck C.A.P. (2011). A prehistoric mural in Spain depicting neurotropic psilocybe mushrooms?. Econ. Bot..

[4] Prentiss D.W., Morgan F.P. (1895). Anhalonium lewinii (mescal buttons). Ther. Gaz..

[5] Jay M. (2019). Mescaline: A Global History of the First Psychedelic.

[6] Hofmann A. (2019). LSD—My Problem Child.

[7] Grinspoon L., Bakalar J. (1981). The psychedelic drug terapie. Curr. Psychiatr. Ther..

[8] Sercl M., Kovarik J., Jaros O. (1961). Clinical experiences with psilocybin Sandoz. Sb. Ved. Pr. Lek. Fak. Karlov. Univ. V Hradci Kral..

[9] Heimann H. (1962). On the treatment of therapy-resistant neuroses with model psychoses (psilocybin). Schweiz Arch. Neurol. Neurochir. Psychiatr..

[10] Reda G.C., Vella G., Cancrini L., D’Agostino E. (1964). Clinical and psychopathological study of psilocybin. Riv. Sper. Freniatr. Med. Leg. Alien. Ment..

[11] Delay J., Pichot P., Lemperiere T., Quetin A.M. (1959). Therapeutic effect of psilocybin on convulsive neurosis. Ann. Med. Psychol..

[12] Duche D.J. (1961). The effects of psilocybin in a case of hysteria. Sem. Hop..

[13] Růzicková R., Bílý D., Vyhnánková M., Dubanský B., Konias V., Soucek Z. (1967). Effect of psilocybine in chronic schizophrenias. I. Clinical findings. Ceskoslov. Psychiatr..

[14] Fisher G. (1970). The psycholytic treatment of a childhood schizophrenic girl. Int. J. Soc. Psychiatry.

[15] Mentzner R., Mentzer R. (2005). Sacred Mushroom of Visions: Teonanacatl: A Sourcebook on the Psilocybin Mushroom.

[16] Keeler M.H., Reifler C.B. (1967). Suicide during an LSD reaction. Am. J. Psychiatr..

[17] Rucker J.J.H., Iliff J., Nutt D.J. (2018). Psychiatry & psychedelic drugs. Past, present & future. Neuropharmacology.

[18] Tyls F., Palenicek T., Horacek J. (2014). Psilocybin- Summary of knowledge and new perspectives. Eur. Neuropsychopharmacol..

[19] Cody J.T., Bogusz M.J., Science B.V. (2008). Chapter 4 Hallucinogenes. Handbook of Analytical Separations.

[20] StiJve T. (1995). Worldwide occurrence of psychoactive mushrooms-an update. Czen Mycol..

[21] Derosa G., Maffioli P. (2014). Alkaloids in the nature: Pharmacological applications in clinical practice of berberine and mate tea. Curr. Top. Med. Chem..

[22] Tittarelli R., Mannocchi G., Pantano F., Romolo F.S. (2015). Recreational use, analysis and toxicity of tryptomines. Curr. Neuropharmacol..

[23] Cayman Chemicals Psilocybin. https://www.caymanchem.com/product/14041/psilocybin.

[24] Ballesteros S., Ramon M.F., Iturralde M.J., Martinez-Arrieta R., Cole S.M. (2006). Natural source of drugs of abuse: Magic mushrooms. New Research on Street Drugs.

[25] Pendersen-Bjergaard S., Sannes E., Rasmussen K.E., Tonnesen F. (1997). Determination of psilocybin in *Psilocybe semilanceata* by capillary zone electrophoresis. J. Chromatogr. Biomed. Sci. Appl..

[26] van Amsterdam J., Opperhuizen A., van den Brink W. (2011). Harm potential of magic mushroom use: A review. Regul Toxicol. Pharmacol..

[27] Hopf A., Eckert H. (1974). Distribution patterns of 14C-psilocin in the brains of various animals. Act. Nerv. Supp..

[28] Law F.C.P., Poon G., Chui Y.C., He S.H. (2014). 14C-Psilocin tissue distribution in pregnant rats after intravenous administration. Funct. Foods Health Dis..

[29] Hasler F., Bourquin D., Brenneisen R., Bär T., Vollenweider F.X. (1997). Determination of psilocin and 4-hydroxyindole-3-yl-acetic-acid in plasma by HPLC-ECD and pharmacokinetic profiles of oral and intravenous psilocybin in man. Pharm. Acta Helv..

[30] Hasler F., Bourquin D., Brenneisen R., Vollenweider F.X. (2002). Renal excretion profiles of psilocin following oral administration of psilocybin: A controller study in man. J. Pharm. Biomed. Anal..

[31] Eivindvik K., Rasmussen K.E., Sund R.B. (1989). Handling of psilocybin and psilocin by everted sacs of rat jejunum and colon. Acta Pharm. Nord..

[32] Passie T., Seifert J., Schneider U., Emrich H.M. (2002). The pharmacology of psilocybin. Addict. Biol..

[33] Helsley S., Fiorella D., Rabin R.A., Winter J.C. (1998). A comparison of N,N-dimethyltryptamine, harmaline, and selected congeners in rats trained with LSD as a discriminative stimulus. Prog. Neuro-Psychopharmacol. Biol. Psychiatry.

[34] Kalberer F., Kreis W., Rutschmann J. (1962). The fate of psilocin in the rat. Biochem. Pharmachol..

[35] Lindenblatt H., Kramer E., Holzmann-Erens P., Gouzoulis-Mayfrank E., Kovar K.A. (1998). Quantitation of psilocin in human plasma by high-performance liquid chromatography and electrochemical detection: Comparison of liquid-liquid extraction with automated on-line solid-phase extraction. J. Chromatogr. B Biomed. Sci. Appl..

[36] Martin R., Schurenkamp J., Gasse A. (2013). Determination of psilocin, bufotenine, LSD, and its metabolites in serum, plasma and urine by SPE-LC-MS/MS. Int. J. Legal Med..

[37] Grieshaber A.F., Moore K.A., Levine B. (2001). The detection of psilocin in human urine. J. Forensic Sci..

[38] Sticht G., Kaferstein H. (2000). Detection of psilocin in body fluids. Forensic Sci. Int..

[39] Manevski N., Kurkela M., Hoglund C., Mauriala T., Court M.H., Yli-Kauhaluoma J., Finel M. (2010). Glucuronidation of psilocin and 4-hydroxyindole by the human UDP-glucuronosyltransferases. Drug Metab. Dispos..

[40] Kovacic P. (2009). Unifying electron transfer mechanism for psilocybin and psilocin. Med. Hypotheses.

[41] Nichols D.E. (2004). Hallucinogens. Pharmacol. Ther..

[42] Ray T.S. (2010). Psychedelics and the human receptorome. PLoS ONE.

[43] Creese I., Burt D.R., Snyder S.H. (1975). The dopamine receptor: Different binding of d-LSD and related agents to agonist and antagonist states. Life Sci..

[44] Vollenweider F.X., Vollenweider-Scherpenhuysen M.F.I., Babler A., Vogel H., Hell D. (1998). Psilocybin induces schizophrenia-like psychosis in humans via serotonin-2 agonist action. Neuroreport.

[45] Vollenweider F.X., Vontobel P., Hell D., Leenders K.L. (1999). 5-HAT modulation of dopamine release in basal ganglia in psilocybin-induced psychosis in man-a PET study with [11C] raclopride. Neuropsychopharmacology.

[46] McKenn D.J., Repke D.B., Lo L., Peroutka S.J. (1990). Differential interactions of indolealkylamines with 5-hydroxytryptamine receptor subtypes. Neuropharmacology.

[47] Horita A. (1963). Some biochemical studies on psilocybin and psilocin. J. Neuropsychiatr..

[48] Fink M. (1969). EEG and human psychopharmacology. Annu. Rev. Pharmacol..

[49] Horibe M. (1974). The effects of psilocybin on EEG and behaviour in monkeys. Act. Nerv. Super..

[50] Meldrum B.S., Naquet R. (1971). Effects of psilocybin, dimethyl-tryptamine, mescaline and various lysergic acid derivatives on the EEG and on photically induced epilepsy in the baboon (Papio papio). Electroencephalogr. Clin. Neurophysiol..

[51] Da Fonseca J.S., Cardoso C., Salgueiro E., Fialho M.L. (1965). Neuro-physiological and psychological study of psilocybin-induced modification of visual information processing in man. Neuro Psychopharmacol..

[52] Rynearson R.R., Wilson M.R., Bickford R.G. (1968). Psilocybin-induced changes in psychologic function, electroencephalogram, and light-evoked potentials in humans ubjects. Mayo Clin. Proc..

[53] Kometer M., Schmidt A., Jäncke L., Vollenweider F.X. (2013). Activation of serotonin 2A receptors underlies the psilocybin-induced effects on α oscillations, N170 visual-evoked potentials, and visual hallucinations. J. Neurosci..

[54] Muthukumaraswamy S.D., Carhart-Harris R.L., Moran R.J., Brookes M.J., Williams T.M., Errtizoe D., Sessa B., Papadopoulos A., Bolstridge M., Singh K.D. (2013). Broadband Cortical Desynchronization Underlies the Human Psychedelic State. J. Neurosci..

[55] Vollenweidera F.X., Leendersb K.L., Scharfettera C., Maguire P., Stadelmann O., Angsta J. (1997). Positron emission tomography and fluorodeoxyglucose studies of metabolic hyperfrontality and psychopathology in the psilocybin model of psychosis. Neuropsychopharmacology.

[56] Gouzoulis-Mayfrank E., Schreckenberger M., Sabri O., Arning C., Thelen B., Spitzer M., Kovar K.A., Hermle L., Büll U., Sass H. (1999). Neurometabolic effects of psilocybin, 3, 4 methylenedioxyethylamphetamine (MDE) and d-methamphetamine in healthy volunteers. A double-blind, placebo-controlled PET study with [18F]FDG. Neuropsychopharmacology.

[57] Carhart-Harris R.L., Erritzoe D., Williams T., Stone J.M., Reed L.J., Colasanti A., Tyacke R.J., Leech R., Malizia A.L., Murphy K. (2012). Neural correlates of the psychedelic state as determined by fMRI studies with psilocybin. Proc. Natl. Acad. Sci. USA.

[58] Lewis C.R., Preller K.H., Kraehenmann R., Michels L., Staempfli P., Vollenweider F.X. (2017). Two dose investigation of the 5-HT-agonist psilocybin on relative and global cerebral blood flow. NeuroImage.

[59] Carhart-Harris R.L., Leech R., Williams T.M., Erritzoe D., Abbasi N., Bargiotas T., Hobden P., Sharp D.J., Evans J., Feilding A. (2012). Implications for psychedelic-assisted psychotherapy: Functional magnetic resonance imaging study with psilocybin. Br. J. Psychiatry.

[60] Madsen M.K., Fisher P.M., Burmester D., Dyssegaard A., Stenbæk D.S., Kristiansen S., Johansen S.S., Lehel S., Linnet K., Svarer C. (2019). Psychedelic effects of psilocybin correlate with serotonin 2A receptor occupancy and plasma psilocin levels. Neuropsychopharmacology.

[61] Stenbæk D.S., Madsen M.K., Ozenne B., Kristiansen S., Burmester D., Erritzoe D., Knudsen G.M., Fisher P.M. (2021). Brain serotonin 2A receptor binding predicts subjective temporal and mystical effects of psilocybin in healthy humans. J. Psychopharmacol..

[62] Madsen M.K., Fishera P.M., Stenbæka D.S., Kristiansen S., Burmester D., Lehelc S., Páleníček T., Kuchařde M., Svarera C., Ozenne B. (2020). A single psilocybin dose is associated with long-term increased mindfulness, preceded by a proportional change in neocortical 5-HT2A receptor binding. Eur. Neuropsychopharmacol..

[63] Garg U., Knoblauch J., FrazeeII C., Baron A., Dudley M., Ketha H., Garg U. (2020). Accidental death involving psilocin from ingesting “magic mushroom”. Toxicology Cases for the Clinical and Forensic Laboratory.

[64] Gerault A., Picart D. (1996). Intoxication mortelle a la suite de la consommation volontaire et en groupe de champignons hallucinogenes. Bull. Soc. Mycol. Fr..

[65] PubChem Psilocyn. https://pubchem.ncbi.nlm.nih.gov/compound/10624#section=Acute-Effects&fullscreen=true.

[66] PubChem Psilocin. https://pubchem.ncbi.nlm.nih.gov/compound/4980#section=Hazard-Classes-and-Categories.

[67] Harris I., Wolbach A.B., Wikler A., Miner E.J. (1961). Cross tolerance between LSD and psilocybin. Psychopharmacologia.

[68] Geyer M.A., Vollenweider F.X. (2008). Serotonin research: Contributions to under standing psychoses. Trends Pharmacol. Sci..

[69] Hasler F., Grimberg U., Benz M.A., Huber T., Vollenweider F.X. (2004). Acute psychological and physiological effects of psilocybin in healthy humans: A double-blind placebo-controlled dose effect study. Psychopharmacology.

[70] Hollister L.E. (1961). Clinical, biochemical and psychologic effects of psilocybin. Arch. Int. Pharmacodyn. Ther..

[71] Halpern J.H. (2004). Hallucinogenes and dissociative agents naturally growing in the United States. Pharmacol. Ther..

[72] Denis-Oliveira R.J. (2017). Metabolism of psilocybin and psilocin: Clinical and forensic toxicological relevance. Drug Metab. Rev..

[73] Fowler J.S., Volkow N.D., Wang G.J., Pappas N., Logan J., MacGregor R., Alexoff D., Shea C., Schlyer D., Wolf A.P. (1996). Inhibition of monoamine oxidase B in the brains of smokers. Nature.

[74] Austin E., Myronc H.S., Summerbell R.K., Mackenzie C.A. (2019). Acute renal injury cause by confirmed *Psilocybe cubensis* mushroom ingestion. Med. Mycol. Case Rep..

[75] Peden N., Bisset A.F., Macaulay K.E.C., Crooks J. (1981). Clinical toxicology of ‘magic mushroom’ ingestion. Postgrad. Med. J..

[76] Johnson M., Richards W., Griffiths R. (2008). Human hallucinogen research: Guideline for safety. J. Psychopharmacol..

[77] Lewis C.R., Preller K.H., Braden B.B., Riecken C., Vollenweider F.X. (2020). Rostral anterior cingulate thickness predicts the emotional psilocybin experience. Biomedicines.

[78] Strassman R.J. (1984). Adverse reactions to psychedelic drugs. A review of the literature. J. Ment. Dis..

[79] van Went G.F. (1978). Mutagenicity testing of 3 hallucinogenes: LSD, psilocybin and delta-9-THC, using the micronucleus test. Experientia.

[80] Grob C.S., Danforth A.L., Chopra G.S., Hagerty M., McKay C.R., Halberstadt A.L., Greer G.R. (2011). Pilot study of psilocybin treatment for anxiety in patients with advanced-stage cancer. Arch. Gen. Psychiatry.

[81] Griffiths R.R., Johnson M.W., Carducci M.A., Umbricht A., Richards W.A., Richards B.D., Cosimano M.P., Klinedinst M.A. (2016). Psilocybin produces substantial and sustained decreases in depression and anxiety in patients with life-threatening cancer: A randomized double-blind trial. J. Psychopharmacol..

[82] Ross S., Bossis A., Guss J., Agin-Liebes G., Malone T., Cohen B., Mennenga S.E., Belser A., Kalliontzi K., Babb J. (2016). Rapid and sustained symptom reduction following psilocybin treatment for anxiety and depression in patients with life-threatening cancer: A randomized controlled trial. J. Psychopharmacol..

[83] Carhart-Harris R.L., Bolstridge M., Rucker J., Day C.M.J., Erritzoe D., Kaelen M., Bloomfield M., Rickard J.A., Forbes B., Feilding A. (2016). Psilocybin with psychological support for treatment-resistant depression: An open-label feasibility study. Lancet Psychiatry.

[84] Carhart-Harris R.L., Bolstridge M., Day C.M.J., Rucker J., Watts R., Erritzoe D., Kaelen M., Giribaldi B., Bloomfield M., Pilling S. (2018). Psilocybin with psychological support for treatment-resistant depression: Six-month follow-up. Psychopharmacology.

[85] Davis A.K., Barrett F.S., May D.G., Cosimano M.P., Sepeda N.D., Johnson M.W., Finan P.H., Griffiths R.R. (2021). Effects of psilocybin-Assisted therapy on major depressive disorder. A randomized clinical trial. JAMA Psychiatry.

[86] Carhart-Harris R., Giribaldi B., Watts R., Baker-Jones M., Murphy-Beiner A., Murphy R., Martell J., Blemings A., Erritzoe D., Nutt D. (2021). Trial of psilocybin versus escitalopram for depression. Trial of psilocybin versus escitalopram for depression. N. Engl. J. Med..

[87] Moreno F.A., Wiegand C.B., Taitano E.K., Delgado P.L. (2006). Safety, tolerability, and efficacy of psilocybin in 9 patients with obsessive-compulsive disorder. J. Clin. Psychiatry.

[88] Wilcox J.A. (2014). Psilocybin and obsessive compulsive disorder. J. Psychoact. Drugs.

[89] Leonard H.L., Rapoport J.L. (1987). Relief of obsessive-compulsive symptoms by LSD and psilocin. Am. J. Psychiatry.

[90] Bogenschutz M.P., Forcehimes A.A., Pommy J.A., Wilcox C.E., Barbosa P.C.R., Strassman R.J. (2015). Psilocybin assisted treatment for alcohol dependance: A proof-of-concept study. J. Psychopharmacol..

[91] Johnson M.W., Garcia-Romeu A., Cosimano M.P., Griffiths R.R. (2014). Pilot study of the 5-HT2AR agonist psilocybin in the treatment of tobacco addiction. J. Psychopharmacol..

[92] Garcia-Romeu A., Griffiths R.R., Johnson M.W. (2014). Psilocybin-occasioned mystical experiences in the treatment of tobacco addiction. Curr. Drug Abuse Rev..

[93] NIH. U.S. National Library of Medicine ClinicalTrials.gov. https://clinicaltrials.gov/ct2/show/NCT04161066.

[94] Muthukumaraswamy S., Forsyth A., Lumley T. (2021). Blinding and expectancy confounds in psychedelic randomised controlled trials. Expert Rev. Clin. Pharmacol..

